# Tryptophan-2,3-Dioxygenase as a Therapeutic Target in Digestive System Diseases

**DOI:** 10.3390/biology14030295

**Published:** 2025-03-15

**Authors:** Zhengsen Wang, Xianxian Xie, Yu Xue, Yixuan Chen

**Affiliations:** The Engineering Technological Center of Mushroom Industry, Minnan Normal University, Zhangzhou 363000, China

**Keywords:** tryptophan-2,3-dioxygenase (TDO2), digestive system diseases, tryptophan metabolism, kynurenine, treatment strategy

## Abstract

Tryptophan (Trp) is an essential amino acid that must be acquired exclusively through dietary intake. The metabolism of tryptophan plays a critical role in maintaining immune homeostasis and tolerance, as well as in preventing excessive inflammatory responses. Tryptophan-2,3-dioxygenase (TDO2) is a tetrameric heme protein and serves as one of the pivotal rate-limiting enzymes in the first step of tryptophan metabolism. Dysregulation of TDO2 expression has been observed in various digestive system diseases, encompassing those related to the oral cavity, esophagus, liver, stomach, pancreas, and colon and rectum. Digestive system diseases are the most common clinical diseases, with complex clinical manifestations and interrelated symptoms, and have become a research hotspot in the field of medicine. Studies have demonstrated that aberrant TDO2 expression is closely associated with various clinical manifestations and disease outcomes in patients with digestive system disorders. Consequently, TDO2 has garnered increasing recognition as a promising therapeutic target for digestive system diseases in recent years, attracting growing attention. This article provides a brief overview of the role of TDO2 in the tryptophan pathway, emphasizing its significant involvement in diseases of the digestive system. Strategies targeting TDO2 through specific inhibitors suggest considerable promise in enhancing therapeutic outcomes for digestive diseases. Thus, this review concludes by discussing recent advancements in the development of TDO2 inhibitors. We believe that targeted inhibition of TDO2 combined with immunotherapy, the screening of a large number of natural products, and the assistance of artificial intelligence in drug design will be important directions for developing more effective TDO2 inhibitors and improving treatment outcomes in the future.

## Simple Summary

Tryptophan (Trp) is an essential amino acid that must be acquired exclusively through dietary intake. The metabolism of tryptophan plays a critical role in maintaining immune homeostasis and tolerance, as well as in preventing excessive inflammatory responses. Tryptophan-2,3-dioxygenase (TDO2) serves as one of the pivotal rate-limiting enzymes in the first step of tryptophan metabolism. Dysregulation of TDO2 expression has been found in various digestive system diseases, including digestive system cancers and inflammatory digestive system diseases. Consequently, TDO2 has garnered increasing recognition as a promising therapeutic target for digestive system diseases in recent years, attracting growing attention. This review elucidates the mechanisms by which TDO2 functions within the tryptophan metabolic pathway, its role in digestive system diseases, and recent advancements in TDO2 inhibitor research. The objective is to offer novel insights and a comprehensive theoretical foundation for future investigations in tryptophan metabolism.

## 1. Introduction

The digestive system is responsible for the breakdown of food, absorption of nutrients, and elimination of waste. Therefore, the health of the digestive system is closely related to quality of life. However, shifts in lifestyle and dietary patterns have led to an increase in gastrointestinal diseases, making them among the most prevalent clinical conditions. Their multifaceted and interconnected clinical manifestations have positioned them as a focal point for research in the medical field [1].

Diseases of the digestive system can be categorized as either malignant or benign. Malignant digestive diseases encompass gastric cancer, liver cancer, colorectal cancer, oral cancer, esophageal cancer, and others [2]. According to the latest data, gastrointestinal tumors exhibit high incidence and mortality rates, posing a significant threat to human health [3,4] and placing a substantial economic burden on healthcare systems [5]. Most malignant digestive system diseases are typically diagnosed at middle or late stages, leading to poor prognosis and shorter survival periods for patients [6,7]. Benign diseases of the digestive system, such as inflammatory bowel disease (IBD) [8], nonalcoholic fatty liver disease (NAFLD) [9], and benign peptic ulcers [10], are not immediately life-threatening but often recur and are challenging to manage. Without appropriate and timely treatment, these conditions can progress and carry a risk of malignant transformation [11,12,13].

Tryptophan (Trp) is an essential amino acid that must be acquired exclusively through dietary intake. Tryptophan not only plays a pivotal role in protein synthesis but also serves as a precursor to several bioactive compounds, including serotonin, melatonin, and niacin (vitamin B3) [14]. Additionally, the human body maintains immune homeosta and tolerance, as well as avoids acute and chronic excessive inflammatory responses, through the metabolic degradation of tryptophan [15]. Indoleamine-2,3-dioxygenase 1/2 (IDO1/2) and tryptophan-2,3-dioxygenase (TDO2) are key rate-limiting enzymes involved in the first step of tryptophan metabolism, playing a critical role in subsequent metabolic pathways involving tryptophan. IDO1/2 was described to be present in various human organs, including the liver, placenta, lung, small intestine, and so on, whereas TDO2 is primarily expressed in the liver [16,17]. Most previous studies have focused on the role of IDO in cancer [18], including gastric cancer [19], melanoma [20], endometrial cancer [21], acute myeloid leukemia [22], and diffuse large B-cell lymphoma [23]. With the advancement of research, the pivotal role of TDO2 in various cancers and inflammatory diseases has become increasingly evident [24,25,26,27]. Moreover, because TDO2 only works on Trp while IDO1/2 has lower substrate specificity [17], TDO2 has garnered significant attention in recent years as a potentially promising therapeutic target.

Numerous studies have demonstrated that imbalanced expression of TDO2 significantly contributes to the development and progression of digestive system diseases. Several TDO2 inhibitors have been developed. Furthermore, the co-administration of TDO2 inhibitors alongside other immunotherapeutic agents, including anti-programmed cell death ligand 1 (PD-L1) and anti-programmed cell death 1 (PD-1) immune checkpoint inhibitors, have shown promising results in treating diseases associated with TDO2 expression dysregulation [28,29,30]. In this paper, the keywords of TDO2, digestive system, and diseases were searched in the databases of PubMed, Google Scholar, and Web of Science to review the involvement of TDO2 in digestive system diseases. We also offer a succinct overview of TDO2 inhibitors and their current research advancements. Furthermore, we also discuss possible reasons that could lead to difficulties in clinical trials of TDO2 inhibitors. We believe that, in addition to the development of traditional small-molecule drugs, the abundance of natural products in nature and the assistance of artificial intelligence will be important directions for developing more effective TDO2 inhibitors in the future.

## 2. Summary of the Tryptophan Metabolism

### 2.1. Kyn Pathway

**Figure 1:**
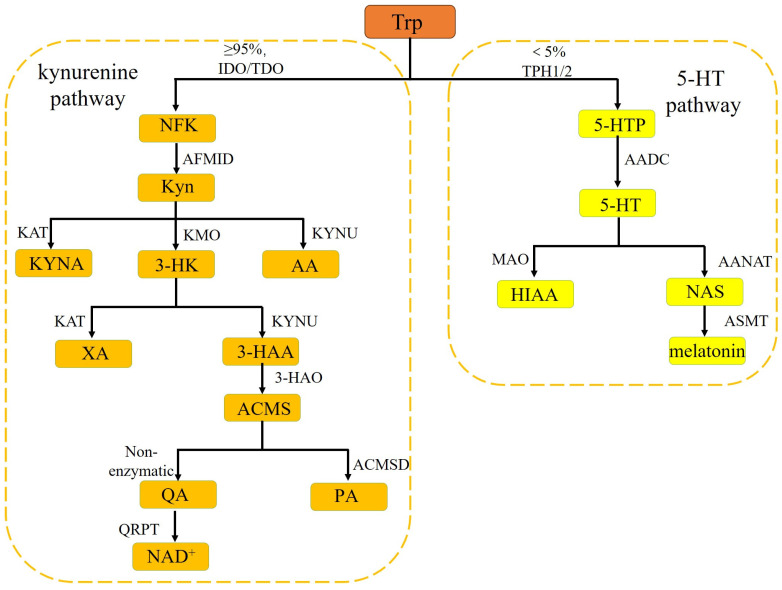
Summary of the Trp metabolism pathway: Kyn pathway and 5-HT pathway. All of the abbreviations are described in the text.

Tryptophan is among the nine indispensable amino acids, and its L-stereoisomers play a crucial role in protein synthesis and the generation of vital molecules, primarily encompassing metabolites of the kynurenine (Kyn) pathway and the production of 5-hydroxytryptamine (5-HT) [14,31]. More than 95% of free tryptophan in the human body is metabolized through the kynurenine (Kyn) pathway, generating metabolites that play important roles in neurotransmission and immune responses regulation [32,33]. Trp is converted into N-formyl-L-kynurenine (NFK) by the enzymes IDO1/2 or TDO2, and then NFK is converted into Kyn by arylformamidase (AFMID). The majority of Kyn is converted to 3-hydroxykyn (3-HK) by enzyme kynurenine 3-monooxygenase (KMO). 3-HK can be transformed into xanthurenic acid (XA) by the enzyme kynurenine aminotransferase (KAT) or converted to 3-hydroxyanthranilic acid (3-HAA) via the enzyme kynureninase (Kynu) catalyzed reaction. 3-HAA undergoes rapid conversion to the unstable 2-amino-3-carboxymuconate-semialdehyde (ACMS) by 3-hydroxyanthraniline 3, 4-dioxygenase (3-HAO), another potent enzyme of the kynurenine pathway. ACMS is metabolized to quinolinic acid (QA) by non-enzymatic conversion. Following this, QA generates NAD^+^ by the action of quinolinate phosphoribosyl transferase (QPRT), or, alternatively, is converted into picolinic acid (PA) via amino-carboxy-muconate-semialdehyde-decarboxylase (ACMSD) [34,35,36]. The remaining small portion of Kyn is further divided into two metabolic pathways. One pathway involves direct conversion to kynurenic acid (Kyna) through the enzymatic action of KAT, while the other pathway leads to the formation of anthranilic acid (AA) via the Kynu metabolic pathway (Figure 1) [34,35].

### 2.2. 5-HT Pathway

Furthermore, a minor fraction of tryptophan undergoes metabolism via the 5-HT pathway. Tryptophan hydroxylase 1 or 2 (TPH1 or TPH2) catalyzes the conversion of tryptophan (Trp) to 5-hydroxytryptophan (5-HTP). Subsequently, 5-HTP undergoes decarboxylation by aromatic amino acid decarboxylase (AADC) to 5-hydroxytryptamine (5-HT), which is mainly converted into 5-hydroxyindoleacetic acid (5-HIAA) by the enzyme monoamine oxidase (MAO). In addition, 5-HT can be further metabolized by arylalkylamine N-acetyltransferase (AANAT) into N-acetylserotonin (NAS), which is ultimately converted into melatonin by N-acetylserotonin O-methyltransferase (ASMT) (Figure 1) [37,38,39].

## 3. TDO2, the Key Rate-Limiting Enzyme in the Tryptophan Metabolic Pathway

### 3.1. The Evolution of TDO2

TDO2, also known as TDO, was initially identified in the liver of rats in 1936 [40]. Subsequent investigations have demonstrated its ubiquitous expression across many species, from mammalian to invertebrate. In insects, TDO2 is also known as Drosophila bright-red-eye-color gene [41]. TDO2 has also been found to be expressed in yeast [42], *Pseudomonas fluorescens* [43], and *Bacillus brevis* [44]. However, its expression has not been detected in fungi [45]. Gene sequencing has revealed significant sequence homology between bacterial TDO2 and human TDO2 (hTDO2), indicating strong conservation of the TDO2 gene [45]. In humans, TDO2 is located on chromosome 4q32.1, comprising 12 exons and 11 introns, totaling 65,699 base pairs and encoding 406 amino acids [46]. TDO2 is predominantly expressed in the liver, but it can also be detected in the kidneys [47], skin [48], brain [49], and pregnant uterus [50] after certain stimuli.

### 3.2. The Structure of TDO2

TDO2 is a tetrameric heme protein with 35–45 kD per monomer [51]. The N-terminal residues of each monomer contribute to the substrate-binding site in adjacent monomers, thus defining the structure of TDO2 as a dimer of dimers [52,53]. The hTDO2 monomer exhibits a complete alpha-helical structure, named αA to αL. The interface of the hTDO tetramer has three long helices, αB, αC, and αJ, each with 6–10 turns. Additionally, a long helix is formed by the combination of αE and αH, contributing to the formation of a tetrameric bundle in each subunit. The heme is situated at one end of the four-helical bundle, with its proximal His328 ligand originating from the C-terminal region of helix αJ. Furthermore, the fragment consisting of helix–loop–helix elements (αH_1_-αH_2_) is located in close proximity to the opposite end of this bundle [54].

### 3.3. The Process of TDO2 Catalyzing the Degradation of Tryptophan

TDO2 catalyzes the conversion of L-tryptophan to N-formylkynurenine (NFK) through the binding of two oxygen atoms from oxygen molecules to the indole portion of tryptophan, a process referred to as the two-step ferryl-based deoxygenation mechanism [55,56,57]. The initial step entails the binding of the heme-iron-bound dioxygen to the C_2_ of L-Trp via a 2- indolenylperoxo transition state, resulting in the formation of a ferryl and Trp-epoxide intermediate. The second step involves the protonation of the epoxide by the ammonium ion of L-Trp, leading to the opening of the epoxide ring and initiation of ferryl-oxygen bonding to C_2_. This ultimately results in the cleavage of the C_2_-C_3_ bond and formation of the NFK product. Afterwards, the formyl group of NFK binds to the iron atom, leading to disruption of the JK loop. Due to the disorder of the JK loop, the NFK active site is released, allowing L-Trp to bind again and initiate the next cycle of reactions [54].

## 4. The Role of TDO2 in Digestive System Diseases

**Figure 2:**
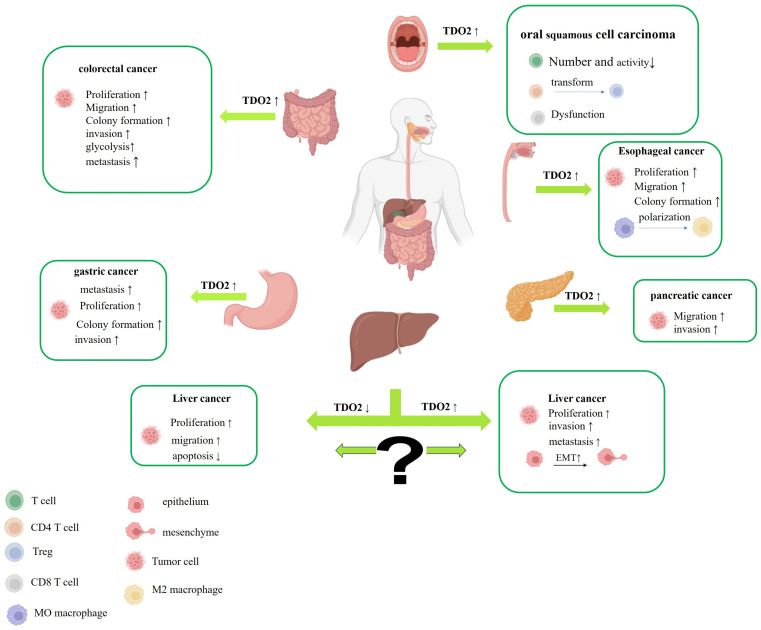
Schematic abstract of the intimate association between TDO2 and malignant digestive system diseases presented in this review. Briefly, dysregulation of TDO2 expression has been observed in various malignant diseases affecting the digestive system, encompassing those related to the oral cavity, esophagus, liver, stomach, pancreas, and colon and rectum. The upward arrows signify a promoting effect, whereas the downward arrows denote an inhibitory effect. Illustration created with BioRender.com.

**Figure 3:**
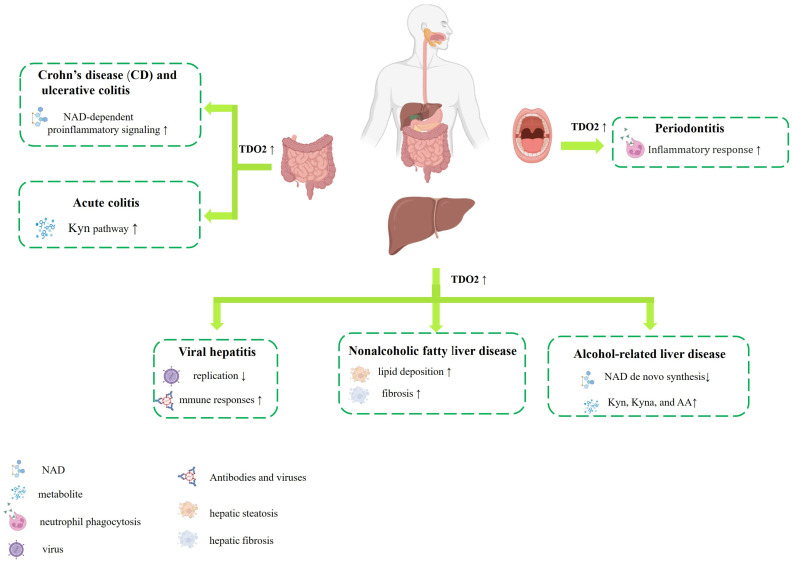
Schematic abstract of the intimate association between TDO2 and benign digestive system diseases presented in this review. Dysregulation of TDO2 expression has been observed in various benign diseases affecting the digestive system, encompassing periodontitis, inflammatory bowel disease, and liver disorders. The upward arrows signify a promoting effect, whereas the downward arrows denote an inhibitory effect. Illustration created with BioRender.com.

Trp metabolism is an important biological pathway in the human body, playing a crucial role in maintaining the health of the digestive system. As one of the key rate-limiting enzymes in the tryptophan metabolic pathway, imbalanced TDO2 expression significantly contributes to the occurrence and development of digestive system diseases, which are summarized in Table 1 and Table 2. Aberrant TDO2 expression has been observed in numerous malignant tumors of the digestive system (Figure 2) (Table 1). The metabolite Kyn, produced from Trp under TDO2 catalysis, is believed to be a critical factor in suppressing T-cell proliferation and inducing T-cell apoptosis [58,59,60]. Meanwhile, Kyn serves as a crucial endogenous ligand for the aromatic hydrocarbon receptor (AhR) [61]. Upon activation by ligands, AhR can translocate to the nucleus, which further enhances TDO2 activity, leading to the deregulation of cell–cell contact, inducing unbalanced proliferation and differentiation of cells, ultimately fostering the development of tumors [62,63]. Furthermore, evidence indicates that the overexpression of TDO2 plays a significant role in inflammatory diseases of the digestive system, including periodontitis [64], inflammatory bowel disease [65], and liver disorders [66,67,68] (Figure 3) (Table 2). Therefore, investigating the mechanism of TDO2 in digestive system diseases not only holds significant implications for improving patient outcomes and enhancing their quality of life, but also offers a novel perspective for exploring innovative approaches to the treatment and prevention of digestive system diseases.

### 4.1. TDO2 and Malignant Digestive System Diseases

#### 4.1.1. TDO2 and Oral Squamous Cell Carcinoma

Oral squamous cell carcinoma (OSCC) is a malignant tumor originating in the oral epithelium and is the main type of head and neck squamous cell carcinoma (HNSCC). OSCC impacts over 300,000 individuals globally annually [69,70]. OSCC patients are typically diagnosed at an advanced stage, and exhibit a high mortality rate and poor prognosis [71]. The upregulation of TDO2 expression in OSCC samples suggests a potentially significant role for TDO2 in the occurrence and progression of OSCC [72]. According to the single-cell transcriptomic landscape of precancerous and cancerous tissues in oral carcinogenesis, a subset of myofibroblasts expressing TDO2 was found, located distantly from the tumor nests, with CD4 and CD8 T cells enriched around them. Functional experiments demonstrated that TDO2-expressing myofibroblasts were capable of chemotaxis toward T cells, inducing conversion of CD4 T cells into Tregs and causing functional impairment in CD8 T cells. Treatment with LM10, the inhibitor of TDO2, alleviated the inhibitory states of T cells, restored T cell antitumor response, and prevented malignant progression of OSCC in mouse models [73]. Manar et al. investigated the immuno-oncologic (IO) signature at the surgical tumor margin (TM) of OSCC undergoing malignant transformation and observed a significant upregulation of TDO2 in the tumor compared to TM, along with a significant downregulation of CD8 expression in tumor cells compared to TM. The above data suggest that the increase in TDO2 protein activity and subsequent production of Kyn may play a role in suppressing the antitumor immune response in OSCC by reducing the number and activity of T cells [74].

#### 4.1.2. TDO2 and Esophageal Cancer

Esophageal cancer (ESCA) is a common malignant tumor, with esophageal squamous cell carcinoma (ESCC) being the main subtype of ESCA. It results in over 400,000 fatalities globally annually [75,76] and exhibits a dismal prognosis, with a 5-year survival rate of only 22% [77]. Knockdown of TDO2 expression in human esophageal cancer cell lines TE-10 and TE-11 reduces the number and size of spheroid colonies, inhibits cell proliferation, and induces inactivation of the epidermal growth factor receptor signaling pathway [78]. Additionally, there is a report suggesting that TDO2 may affect the development of ESCC by influencing the polarization of macrophages in the tumor microenvironment (TME). The TME plays a crucial role in cancer development and metastasis. Among the immune cells present in the TME, macrophages are thought to consist of tumor-suppressive (M1) and tumor-supportive (M2) phenotypes. The accumulation of M2 macrophages is linked to unfavorable clinical outcomes and serves as an inhibitory factor in inflammatory responses within solid tumors, including ESCC [79,80,81]. A study revealed that the overexpression of TDO2 in ESCC results in heightened phosphorylation of AKT and GSK3β, thereby facilitating the upregulation of IL-8 expression and polarization of macrophages towards the M2 phenotype, ultimately driving the malignant progression of ESCC [82].

#### 4.1.3. TDO2 and Primary Liver Cancer

Primary liver cancer, commonly known as PLC, ranks as the sixth most prevalent cancer globally and third in terms of mortality among all malignant tumors [83]. PLC is mainly composed of hepatocellular carcinoma (HCC) and intrahepatic cholangiocarcinoma (ICC), with HCC accounting for 75–85% of cases [84]. Liver cancer is an aggressive disease with a high mortality, recurrence rate, and tendency to metastasize to distant sites, resulting in poor overall survival. Currently, there are no effective treatment interventions [85].

There is still considerable debate regarding the role of TDO2 in liver cancer. One perspective posits that excessive TDO2 promotes the progression of liver cancer and exhibits a positive correlation with poor prognosis. It has been reported that the expression of TDO2 is enhanced at high stage (T2–T4) compared to low stage (T1a and T1b) in the tumor tissue of liver cancer patients. Moreover, both cellular and animal experiments have demonstrated that heightened TDO2 expression in tumor cells results in the release of Kyn, which activates AhR. Subsequent activation of AhR leads to an upregulation of IL-6 secretion, further promoted by the STAT3 and TIM4/NF-κB signaling pathways, ultimately inducing tumor progression [86]. Liao’s team demonstrated that the expression of TDO2 in liver cancer tissues from patients was significantly elevated compared to normal tissues following the analysis of collected clinical samples. Patients exhibiting high levels of TDO2 expression were associated with a poorer prognosis. Further investigations revealed that TDO2 facilitates the migration and invasion of liver cancer cells through the signal transduction pathway involving Wnt5a, a key ligand in the non-canonical Wnt signaling pathway [87]. Epithelial-to-mesenchymal transition (EMT) is a critical process that occurs during the early stages of cancer metastasis [88,89]. Xu et al. demonstrated that in HCC cells, TDO2 facilitates the epithelial-to-mesenchymal transition (EMT) process by activating the Kyn-AhR pathway, thereby enhancing HCC metastasis and invasion [90]. MiRNA (microRNA) is a category of non-coding small-molecule RNAs that participate in cellular migration during tumor development through interaction with target mRNA [91,92]. Ai et al. demonstrated that miR-126-5p directly interacts with TDO2, leading to elevated expression of TDO2 and increased tryptophan metabolism in cells, ultimately promoting the proliferation, invasion, and migration of HCC cells [93]. However, the role and expression levels of different miRNAs in tumors vary, and even the same miRNA may exhibit variability within tumors of different types or stages [94]. For example, miR-140-5p and miR-4738-3p suppress the expression of TDO2 by directly binding to the 3′ UTR of TDO2 mRNA [87,95]. Circular RNAs (circRNAs) represent a novel class of endogenous non-coding RNAs that are widely expressed in various tumor tissues such as liver cancer, gastric cancer, and breast cancer, and play a regulatory role in mammalian gene expression [96,97,98]. Natural endogenous circRNAs possess selectively conserved miRNA target sites, thereby functioning as “miRNA sponges” that competitively bind to miRNAs to regulate post-transcriptional activity, or interact with RNA polymerase II in the nucleus to modulate the transcription process [99,100,101]. It has been reported that there is a significant upregulation of CircZNF566 in HCC cell lines and tissues, correlating with a poor prognosis in HCC patients. Functioning as a potent miR-4738-3p sponge in HCC, CircZNF566 alleviates the inhibitory impact of miR-4738-3p on TDO2, thereby enhancing TDO2 expression in tumor cells and fostering the progression and metastasis of HCC [95]. Following the introduction of the Mendelian randomization (MR) concept [102], independent-sample MR, two-sample MR, multivariable MR, and bidirectional MR have assumed an increasingly pivotal role in elucidating the causal relationships between risk factors and diseases [103,104,105]. A recent study utilized the Mendelian randomization (MR) approach to comprehensively investigate the causal relationships between 4719 blood proteins, 21 amino acids, and the risk of PLC, among which TDO2 was identified as a potential biomarker for diagnosing PLC [106].

However, it is noteworthy that certain research findings have yielded contrasting conclusions regarding the involvement of TDO2 in liver cancer development. Notably, a bioinformatics analysis of liver cancer-related data from the public gene expression database (GEO) has indicated that TDO2 expression is decreased in liver cancer tissues [107]. Similarly, there is also an article that points out that in the HCC samples they collected, the expression level of TDO2 in tumor tissue was lower than that in normal tissue. In HCC cell lines, overexpression of TDO2 induced cell cycle arrest by upregulating p21 and p27 [108]. Long non-coding RNA (lncRNA) plays a crucial role in regulating tumorigenesis [109,110,111]. Small nucleolar RNA host gene 17 (SNHG17) is an 1186nt long non-coding RNA (lncRNA) that has been found to be markedly upregulated in HCC tissues and cell lines. It has been reported that SNHG17 promotes HCC cell proliferation and migration, while inhibiting HCC cell apoptosis. Knockdown of SNHG17 in HCC cell lines resulted in the upregulation of TDO2, whereas overexpression of SNHG17 led to a downregulation of TDO2. This result suggests a potential role for TDO2 in inhibiting the progression of HCC; however, it was based on RNA sequencing and bioinformatics analysis, with only simple qPCR verification, lacking direct validation through in vivo or additional in vitro experiments [112].

#### 4.1.4. TDO2 and Pancreatic Cancer

Pancreatic cancer (PC) represents a highly aggressive form of malignant tumor in humans, characterized by an overall 5-year survival rate of approximately 10% [113]. After conducting data analysis using the Cancer Genome Atlas (TCGA), it was determined that IDO1 and TDO2 are commonly co-expressed in patients with PC. Furthermore, the expression levels of both IDO1 and TDO2 exhibit a negative correlation with patients’ survival time, indicating that the combined presence of IDO1 and TDO2, rather than either alone, serves as an independent prognostic indicator for PC. In KPIC orthotopic PC mice, inhibition of the IDO1/TDO2-Kyn-AhR pathway resulted in delayed tumor growth, suppressed tumor metastasis, and enhanced tumor cell apoptosis. What is noteworthy is that treatment with 1-L-MT, a selective inhibitor of IDO1, demonstrated limited therapeutic efficacy in KPIC orthotopic PC mice. These results suggest that TDO2 seems to play a more critical role in the development of PC, and inhibiting both IDO1 and TDO2 may become a new target for immunotherapy of PC [114]. Pancreatic ductal adenocarcinoma (PDAC) represents the most prevalent form of PC [115,116]. IL2RA, also known as CD25, is the alpha chain of the interleukin-2 receptor complex, and it is positively correlated with the poor prognosis of PDAC [117]. Subsequent studies have further established a close association between TDO2 expression and the development of pancreatic cancer. Schwann cells are resident glial cells in the peripheral nervous system that play a significant role in the progression of certain solid tumors, modulating tumor invasiveness and facilitating EMT [118,119,120]. Previously, Schwann cells have been shown to interact with antigens on PDAC tumor cells and promote peripheral nerve invasion in pancreatic cancer [121]. Further studies have demonstrated that PVT1, expressed by tumor-associated nonmyelinating Schwann cells (TASc) in PDAC, promotes PDAC development by regulating TDO2 phosphorylation and the metabolism of kynurenine [122]. Nevertheless, additional clinical samples and experimental data are required to further elucidate the role of TDO2 in PC progression.

#### 4.1.5. TDO2 and Gastric Cancer

Gastric cancer (GC) is one of the most common cancers, ranking fifth in terms of incidence and mortality worldwide [83]. The prognosis for patients with advanced gastric cancer is poor, with a 5-year overall survival rate ranging from 16% to 34% [123,124]. Previous research has demonstrated that excessive activation of AhR in mice exacerbates gastric cancer, suggesting a significant role for AhR in the pathogenesis of gastric cancer [125]. In 2022, Martine et al. reported a novel finding that the expression of AhR was markedly elevated in the tumor tissue of gastric cancer patients, accompanied by a significant upregulation of TDO2 expression [126]. Suppressing immune checkpoints (ICs) can lead to antitumor activation of the immune system, making it one of the most promising approaches for cancer immunotherapy [127]. Danzan et al. identified the expression levels of 10 IC genes including TDO2 in the early stages of GC development and during metastasis by analyzing paired stomach tissue samples (tumor tissue and morphologically normal tissue from the same stomach). The authors believe that the expression of TDO2 is closely related to the metastasis of gastric cancer, with TDO2 expression being four times higher in metastatic tumors compared to non-metastatic tumors. Furthermore, no other IC genes exhibiting a correlation with TDO2 expression were identified in this study. These findings indicate that TDO2 could become an important target for suppressing metastatic gastric cancer [128]. Additionally, there is an article that indicates that the expression of TDO2 is related to the progression of gastric cancer, prognosis, immune infiltration, and the expression of PD-L1, an important immune inhibitory factor on tumor cells. In gastric cancer tissues, the expression of TDO2 is positively associated with the stem cell marker CD44. Knockdown of TDO2 expression inhibits cell proliferation, colony formation, and the invasion of gastric cancer cells, as well as spheroid body formation and the viability of gastric cancer organoid spheres. Therefore, TDO2 expression can serve as an independent prognostic predictor and a potential target gene for precision therapy in gastric cancer [129].

#### 4.1.6. TDO2 and Colorectal Cancer

Colorectal cancer (CRC), a prevalent malignant tumor of the digestive tract, often presents with atypical symptoms in its early stages. By the time of diagnosis, the disease has typically advanced to the intermediate or advanced stage, and currently lacks effective targeted drugs, resulting in a notably low 5-year survival rate [130]. It is estimated that approximately 600,000 individuals die from colorectal cancer globally each year. In developed countries, colorectal cancer ranks second in cancer-related deaths [131]. Hence, colorectal cancer represents a significant menace to human health [132].

Based on the clinical data, TDO2 is overexpressed in the tumor tissue of colorectal cancer patients [24,133]. Cui et al. acquired gene expression profiles of colorectal cancer from the TCGA database and employed single-factor Cox analysis, Lasso regression, and multivariate Cox analysis to identify a six-gene rectal cancer risk model, which includes TDO2. They propose that TDO2 is implicated in amino acid metabolism, and its high expression suggests heightened activity in this pathway, which may unfavorably impact the prognosis of colorectal cancer patients [134]. Subsequently, another study further substantiated this perspective. The analysis of RNA sequencing data and germline whole-genome sequencing data from patients with stage III colorectal cancer, along with their clinical information, revealed a significant correlation between high TDO2 expression and poor prognosis. Consequently, the article concluded that TDO2 represents a potential therapeutic target for colorectal cancer [135]. Overexpression of TDO2 is not only associated with poor prognosis in colorectal cancer, but also related to the clinical stage of colorectal cancer. As the clinical stage advances, the expression level of TDO2 increases correspondingly [136,137]. Moreover, by silencing TDO2 expression in human colon cancer cell lines LoVo and HCT116 using RNAi, the study observed a significant reduction in the proliferation, migration, invasion, and colony formation ability of cells. Concurrently, the levels of KYNU and AhR expression were downregulated to varying extents following TDO2 suppression. Thus, the study proposes that TDO2 modulates colorectal cancer development through the TDO2-KYNU-AhR signaling pathway [136]. Adenomatous polyposis coli (APC) inactivation has been observed in numerous cancer types and is considered a pivotal initiating event in colorectal cancer [138]. It is well known that in normal cells, APC activates glycogen synthase kinase (GSK3β), which subsequently phosphorylates the N-terminal serine/threonine residues of β-catenin, leading to its degradation via ubiquitination. In APC-deficient cancers, β-catenin accumulates due to its impaired degradation. The excessive β-catenin translocates into the nucleus, where it inhibits the activity of the T-cell factor/lymphocyte enhancer factor (TCF/LEF) transcription factor complex, thereby activating the canonical WNT signaling network [139]. It is believed that TDO2 plays a crucial role as a downstream effector in APC-deficient colorectal cancer. The deficiency in APC results in the upregulation of TDO2 gene expression through the TCF4/β-catenin-mediated pathway. Subsequently, TDO2 activates the Kyn-AhR pathway, enhances glycolysis, promotes tumor cell proliferation, and secretes CXCL5 to attract immunogenic tumor-associated macrophages into the tumor microenvironment, thereby suppressing tumor immunity and influencing the progression of colorectal cancer [131]. Colorectal cancer is also prone to distant metastatic lesions, of which liver metastasis is a common type [140]. Toshiaki et al. observed upregulation of Kyn and TDO2 expression in patient-derived colorectal cancer spheroids from metastatic liver lesions. The study further explains that activation of AhR by Kyn, which is generated through the catalysis of TDO2, directly contributes to the promotion of liver metastasis in colorectal cancer. In this process, the activation of PD-L1 is essential. Subsequent analysis of surgical specimens of colon cancers once again confirmed co-expression of TDO2 and PD-L1 in metastatic colorectal cancer, with positively correlated expression levels. Further investigation found that the TDO2-AHR pathway directly regulates the expression of Wnt signal target gene *LGR5* to maintain colorectal cancer stemness and regulate the development of colorectal cancer [141].

**Table 1:** Role and mechanism of TDO2 in malignant digestive system diseases.

Disease Type	Expression of TDO2	Sample	Functions	Mechanisms	Ref.
Oral squamous cell carcinoma	TDO2 ↑	Patient tissue TCGA-HNSC dataset			[72]
Oral squamous cell carcinoma	TDO2 ↑	Patient tissue	Possess the ability to undergo chemotaxis toward T cells but induce the transformation of CD4 T cells into Tregs and cause CD8 T cell dysfunction		[73]
Oral squamous cell carcinoma	TDO2 ↑	Patient tissue HNSCC cell line	Decrease the number and activity of T cells, inhibit antitumor immunity in OSCC		[74]
Esophageal cancer	TDO2 ↑	Patient tissue	Associated with tumor stage, recurrence status, and poor outcome		[78]
Esophageal cancer	TDO2 ↑	Patient tissue ESCC cell lines Mouse tissue	Promote tumor cell proliferation, migration, and colony formation	TDO2/AKT/GSK3*β*/IL-8	[82]
Liver cancer	TDO2 ↑	Patient tissue liver cancer cell lines	Correlated with the poor prognosis, promote tumor cell proliferation	TDO2/Kyn/AhR/IL-6, STAT3 and TIM4/NF-κB	[86]
Liver cancer	TDO2 ↑	Patient tissue, liver cancer cell lines, mouse tissue	Correlated with poor prognosis, promote cancer cell migration and invasion	TDO2/Wnt5a	[87]
Liver cancer	TDO2 ↑	Patient tissue, liver cancer cell lines, orthotopic mouse tissue	Promote the EMT of hepatocellular carcinoma, participate in the metastasis and invasion of HCC	TDO2/Kyn/AhR	[90]
Liver cancer	TDO2 ↑	HCCLM3 cell lines, mouse tissue,	Promote tumor cell proliferation, metastasis, and invasion	miR-126-5p/TDO2 PI3K/AKT and Wnt	[93]
Liver cancer	TDO2 ↑	Patient tissue	Correlated with poor prognosis and promote cell migration, invasion, and proliferation	CircZNF566/miR-4738-3p/TDO2	[95]
Liver cancer	TDO2 ↑	deCODE study, FinnGen Consortium	Used as diagnostic indicators of liver cancer		[106]
Liver cancer	TDO2 ↓	GEO dataset			[107]
Liver cancer	TDO2 ↓	Patient tissue	Correlated with a poor prognosis and adverse clinical outcomes	TDO2/P21, P27	[108]
Liver cancer	TDO2 ↓	GEO, patient tissue cDNA, HCC cell line	Inhibit cell proliferation and migration and promote apoptosis of HCC	SNHG17/ TDO2	[112]
Pancreatic cancer	TDO2 ↑	KPIC cells Mouse tissue	Modulate the migration and invasion of PC cells	TDO2/Kyn/ AhR	[114]
Pancreatic cancer	TDO2 ↑	GEO			[117]
Pancreatic cancer	TDO2 ↑	Patient tissue, mouse tissue	Promote the catalysis of tryptophan to kynurenine and PDAC development	PVT1/p-TDO2	[122]
Gastric cancer	TDO2 ↑	Patient tissue			[128]
Gastric cancer	TDO2 ↑	GEO	Correlated with both progressive disease and clinical outcome		[129]
Colorectal cancer	TDO2 ↑	Patient tissue	Correlated with poor prognosis		[134]
Colorectal cancer	TDO2 ↑	Patient serum, patient tissue	Correlated with poor prognosis		[135]
Colorectal cancer	TDO2 ↑	Patient tissue, CRC cell lines	Associated with the tumor clinical stage in CRC and a poor outcome, promote the proliferation, migration, and invasion abilities as well as colony formation abilitie of cells	TDO2/KYNU/AhR	[136]
Colorectal cancer	TDO2 ↑	Patient tissue, mouse tissue, CRC cell lines	Increase glycolysis to drive anabolic cancer cell growth	TCF4/TDO2/AhR/CXCL5	[131]
Colon cancer (liver metastasis)	TDO2 ↑	Patient tissue, mouse tissue, CRC cell lines	Promote liver metastasis of colon cancer, maintain csc characteristics	TDO2/AhR/ LGR5 (PD-L1)	[141]

Note: The upward arrows indicate an increase in the expression level of TDO2, while the downward arrows represent a decrease in the expression level of TDO2.

### 4.2. TDO2 and Benign Digestive System Diseases

#### 4.2.1. TDO2 and Periodontitis

Periodontitis is a common chronic infectious disease of the oral cavity, characterized by connective tissue matrix degeneration, alveolar resorption, deep periodontal pocket formation, and irreversible inflammation, ultimately leading to tooth loss [142]. The prevalence in developed countries ranges from 30% to 50% [143]. Periodontitis and Crohn’s disease, the latter of which will be discussed later, are both inflammatory conditions with a certain correlation. Evidence indicates that individuals with Crohn’s disease exhibit a heightened prevalence of periodontitis [144]. Following the analysis of differential expressed genes (DEGs) and weighted gene co-expression network analysis (WGCNA) in periodontitis and Crohn’s disease datasets obtained from the Gene Expression Omnibus, a total of 13 crosstalk genes were identified, among which TDO2 was included. The article also indicates that these 13 crosstalk genes are primarily enriched in the interleukin-10 signaling pathway and inflammatory response pathways [64]. Nevertheless, further research is warranted to elucidate the role of TDO2 in periodontitis disease and its precise underlying mechanism [64].

#### 4.2.2. TDO2 and Viral Hepatitis

Viral hepatitis causes more than 1 million deaths globally each year and can lead to cirrhosis and hepatocellular carcinoma. Viral hepatitis is a complex process involving crosstalk of various cell types and a specific cytokine milieu [145], and the role of TDO2 in this process is being explored. For instance, an article suggests that the specific inhibition of TDO2 expression in mice may play a significant role in suppressing mouse hepatitis virus (MHV) infection [146]. Moreover, the expression of TDO2 is related to the metabolic reprogramming of liver cells during viral hepatitis infection. Type I interferon (IFN-I) signals through the ubiquitously expressed IFN1-I receptor (IFNAR1) to induce the expression of interferon stimulated genes (ISGs), which are crucial for the innate immune response and limiting viral replication [147]. During viral infection, the associated genes that suppress tryptophan metabolism in the liver are effectively inhibited, while only TDO2 expression increases. IFN-1 can specifically rewired tryptophan metabolism and promote the oxidation of tryptophan to kynurenine by TDO2, which contributes to regulating redox homeostasis and orchestrates wide-spread reprogramming of central metabolic pathways in hepatocytes [66]. This result indicates that overexpression of TDO2 during viral hepatitis seems to stimulate the immune response and mitigate further disease progression. Nevertheless, the precise role of TDO2 in the context of viral hepatitis warrants further investigation.

#### 4.2.3. TDO2 and Nonalcoholic Fatty Liver Disease

In individuals afflicted with nonalcoholic fatty liver disease (NAFLD), the liver undergoes a series of pathological changes, encompassing simple steatosis, nonalcoholic steatohepatitis (NASH), and cirrhosis [148,149]. The global incidence of NAFLD is escalating, presenting a grave peril to patients’ well-being and imposing substantial economic burdens on both society and affected individuals [150,151]. Data have shown that in mouse models of nonalcoholic steatohepatitis (NASH) induced by hyperlipidemia, TDO2 expression is upregulated in the liver [152]. In the NAFLD model, the expression of hepatic TDO2 exceeds threefold that of the control. Subsequent cell experiments revealed that the absence of TDO2 suppressed NF-κB signaling activity, thereby attenuating the expression levels of liver lipid deposition and fibrosis-related markers induced by palmitate in primary hepatocytes [67]. The data above suggest a strong correlation between TDO2 expression and the pathogenesis of NAFLD.

#### 4.2.4. TDO2 and Alcohol-Related Liver Disease

Alcohol-related liver disease (ALD) is characterized by the accumulation of fat and inflammation resulting from excessive alcohol consumption, which can lead to irreversible damage and fibrosis in liver tissue over time. It is recognized as one of the leading causes of liver disease and liver-related mortality worldwide [153,154,155]. Alcohol exposure disrupts the de novo synthesis of NAD, reduces Trp levels, enhances TDO2 expression, and results in the accumulation of Kyn, Kyna, and AA. However, subsequent investigations have demonstrated that the activation of PPARα, a pivotal enzyme in regulating NAD biosynthesis, may enhance liver alcohol metabolism and modulate cellular catalase activity and NAD levels by further upregulating TDO2 expression, thereby mitigating alcoholic liver injury [68,156].

#### 4.2.5. TDO2 and Inflammatory Bowel Disease

Inflammatory bowel disease (IBD) encompasses ulcerative colitis (UC) and Crohn’s disease (CD), characterized by structural damage of the intestines and persistent, recurrent inflammation [157]. In contemporary society, the prevalence of IBD has been increasing, but the precise etiology and pathophysiology of IBD remain elusive. After detecting the colonic mucosa of UC patients, it was observed that the expression level of TDO2 in the active UC group was significantly elevated compared to both the control group and the remission UC group [65]. However, for UC patients, TDO2 overexpression appears to be a double-edged sword. It has been shown that heightened TDO2 expression is linked to a benign course of UC, characterized by initial activity but prolonged remission lasting more than 5 years [65]. Additionally, through the analysis of publicly available transcriptomic datasets, the investigators observed an upregulation of genes associated with tryptophan metabolism in CD and UC, including KYNU, TDO2, and NAD [158]. In the mouse model of DSS-induced acute colitis, there was a significant increase in hepatic TDO2 expression and an elevation in the Kyn/Trp ratio, indicative of enhanced Trp conversion to Kyn. Furthermore, acute colitis can induce pathological alterations in specific organs, including the brain. Acute enteritis is capable of stimulating the hippocampal region of the brain, leading to a substantial upregulation of TDO2 expression in this area (2.1-fold increase) [159]. The above results strongly indicate the significant involvement of TDO2 in IBD. However, similar to the controversial role of TDO2 in liver cancer, its role in inflammatory bowel disease also appears to be equivocal, necessitating further evidence to elucidate the specific mechanisms of TDO2 in IBD.

**Table 2:** Role and mechanism of TDO2 in benign digestive system diseases.

Disease Type	Expression of TDO2	Sample	Functions	Mechanisms	Ref.
Periodontitis	TDO2 ↑	GEO	Involved in interleukin-10 signaling and inflammatory response		[64]
Viral hepatitis	TDO2 ↑	Mouse tissue Mouse serum primary murine hepatocytes	Correlated with immune responses and viral replication	IFN-I /TDO2	[66]
Nonalcoholic fatty liver disease	TDO2 ↑	Mouse tissue primary murine hepatocytes	Strengthen hepatic lipid deposition and liver fibrosis	TDO2/NF-κB	[67]
Alcohol-related liver disease	TDO2 ↑	Patient tissue, mouse tissue, mouse hepatoma cells	Disrupts NAD de novo synthesis with accumulation of Kyn, Kyna, and AA	PPARα/TDO2	[68]
Ulcerative colitis	TDO2 ↑	patient tissue	Presence of initial activity and then prolong remission for more than 5 years		[65]
Crohn’s disease (CD) and ulcerative colitis	TDO2 ↑	Gene Expression Omnibus (GEO) dataset	Maintain a high level of NAD-dependent proinflammatory signaling		[158]
Acute colitis	TDO2 ↑	mouse tissue, mouse serum	Affect Trp metabolism	Trp/KynKyna	[159]

Note: The upward arrows indicate an increase in the expression level of TDO2.

## 5. Application of Targeting TDO2 in Digestive System Disease Treatment

**Figure 4:**
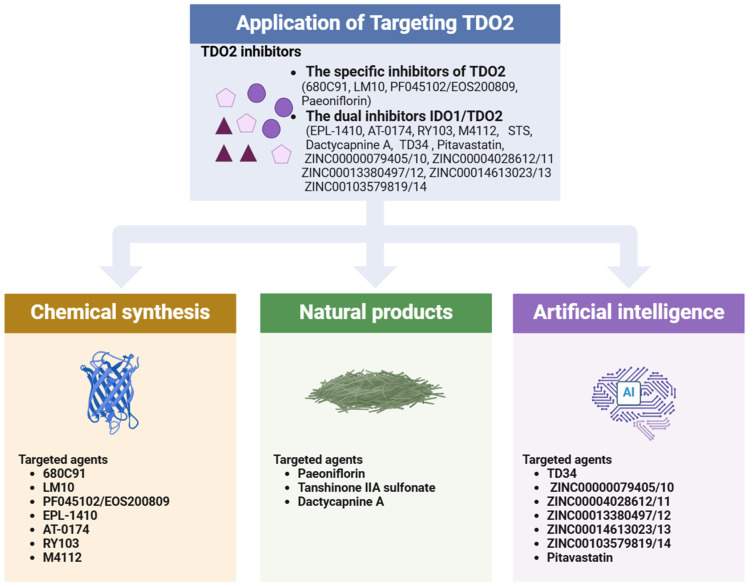
Schematic abstract of recent advances in TDO2 inhibitor studies presented in this review. Illustration Created with BioRender.com.

The initial research on TDO2 initially focused primarily on depression. In comparison to IDO1 inhibitors, which have already entered phase 3 trials, research on TDO2-specific inhibitors has been relatively limited. However, recent research findings strongly indicate the significant roles of TDO2 in tumors and inflammation. Clinical data demonstrate that inhibiting IDO1 alone does not yield satisfactory effects in suppressing disease progression [160]. IDO1 and TDO2 are both key enzymes in the kynurenine pathway, regulating the kynurenine pathway and producing various metabolites, and TDO2 may have a partial compensatory function for IDO1, thus prompting a growing interest in TDO2 inhibitors (only the targeted drugs that have undergone in vivo experiments are summarized in Table 3). This review will present the research findings on TDO2 inhibitors from three key perspectives: traditional chemical synthesis, natural product sources, and artificial intelligence-assisted design (Figure 4).

### 5.1. The Specific Inhibitors of TDO2

#### 5.1.1. TDO2 Specific Inhibitors Derived from Chemical Synthesis

680C91, the first selective inhibitor of TDO2, is a compound featuring an indole skeleton capable of inducing G2 phase arrest in melanoma cell line SK-Mel-28 and human endothelial cells, HUVECs. Furthermore, 680C91 has demonstrated the ability to prompt early apoptosis in human colon adenocarcinoma cell line HCT8 [161]. Hsu et al. noted increased TDO2 expression in fibroblasts adjacent to the implanted murine lung cancer cells within a lung cancer model. Administration of 680C91 led to enhanced T-cell activity, improved dendritic cell (DC) function, and reduced tumor metastasis [162]. Nevertheless, its poor solubility and bioavailability render it unsuitable for clinical application [163]. LM10, which contains a tetrazolyl-vinyl side chain, exhibits high solubility and bioavailability, rendering it an effective TDO2 inhibitor. In a mouse model, LM10 has demonstrated potent antitumor activity against TDO2 with minimal toxicity in mice, positioning it as a promising candidate for targeted TDO2 therapy [164]. Subsequently, the aminoisoxazole series of compounds identified through high-throughput screening demonstrated effective inhibition of TDO2 activity; however, these compounds are not suitable for clinical application due to their instability in whole blood [165]. The compound PF045102/EOS 200809 has a strong inhibitory effect on TDO2, with its inhibitory effect on TDO2 being twice that of 680C91 and 100 times that of LM10. When combined with anti-CTLA-4 or anti-PD-1, it synergistically enhances its antitumor activity. Consequently, the compound PF0 45102/EOS 200809 is considered a promising clinical drug targeting TDO2 [166].

#### 5.1.2. TDO2 Specific Inhibitors Derived from Natural Products

Natural products, renowned for their wide-ranging structures, serve as crucial origins for discovering novel drug leads [167]. Due to the significant challenges in developing high-affinity TDO2 inhibitors, in addition to traditional chemical synthesis, identifying and extracting bioactive compounds from natural products represents a promising alternative approach for discovering TDO2 inhibitors. *Paeonia lactiflora* Pall. (*PaeR*) is an important Chinese medicinal herb that has been widely used in clinics for centuries [168]. Liang et al. demonstrated that *PaeR* extract regulates tryptophan metabolism in depression-like mice by inhibiting TDO2 expression. To identify the active components responsible for this inhibition, they employed high-throughput screening methods, including molecular docking, ligand fishing, and a dual-luciferase reporter assay system, ultimately isolating paeoniflorin from *PaeR* and validating its function. Experimental results showed that paeoniflorin not only significantly suppresses TDO2 expression in HepG2 liver cancer cells overexpressing TDO2 (approximately one-third lower) but also markedly reduces TDO2 expression in major depressive disorder mice [169].

### 5.2. The Dual Inhibitors of IDO1/TDO2

#### 5.2.1. Dual Inhibitors of IDO1/TDO2 Derived from Chemical Synthesis

Moreover, the development of dual inhibitors targeting IDO1/TDO2, which are believed to exert a more potent effect on silencing the kynurenine pathway, has emerged as a prominent area of research. EPL-1410 is a dual inhibitor of IDO1 and TDO2, classified as a fusion heterocyclic analog, exhibiting excellent metabolic stability and safety profiles. Pharmacokinetic studies indicate that EPL-1410 exhibits favorable absolute oral bioavailability in both mice and rats. Furthermore, EPL-1410 demonstrates a pronounced dose-dependent pharmacological effect in mouse models of colon cancer and melanoma, effectively reducing the levels of biomarkers (Kyn/Trp) in plasma, tumor-draining lymph nodes, and tumor tissue without eliciting any treatment-related adverse clinical symptoms or weight loss. These advantages make it a promising candidate for immuno-oncology therapy [170]. AT-0174 can competitively inhibit the binding of IDO1 and TDO2 to tryptophan. In a breast cancer model, AT-0174 demonstrated superior inhibition of tumor growth, macrophage invasion, and PD-L1 expression, and prolonged overall survival compared to IDO1 inhibitors alone [28]. Simultaneously, the combination of AT-0174 with anti-PD-1 therapy significantly enhances the survival rate of mice harboring cisplatin-resistant tumors, demonstrating a two-fold increase compared to the control. Furthermore, AT-0174 exhibits excellent tolerability and non-toxicity towards normal tissue [29]. A recent study revealed that the combination of AT-0174 and temozolomide resulted in a further reduction in tumor growth and improved survival in an orthotopic mouse model of glioblastoma [171]. RY103, a derivative of tryptanthrin, has the ability to inhibit the migration, invasion, and proliferation of the malignancy of gliomas by blocking the IDO/TDO-Kyn-AhR-AQP4 signaling pathway [172]. Similarly, RY103 acts on the Kyn-AhR pathway to inhibit the migration and invasion of PC cells in vitro and improves the immunosuppressive tumor microenvironment in KPIC orthotopic PC mouse models and Pan02 tumor-bearing mice [114]. An article published in 2024 demonstrates that RY103 exhibits anti-angiogenic properties in glioblastoma, and its combination with sunitinib yields enhanced efficacy [173]. M4112, a potent and selective dual inhibitor of IDO1/TDO2, has completed phase 1 trials involving 15 patients with advanced solid tumors. However, the results were not encouraging. Additionally, due to the limitations of the clinical study, tumor biopsies were not obtained from patients, precluding the evaluation of changes in IDO1/TDO2 expression and the tumor microenvironment [174]. Future studies are warranted to investigate the pharmacodynamics, safety, and efficacy of M4112 in combination with checkpoint inhibitors.

#### 5.2.2. Dual Inhibitors of IDO1/TDO2 Derived from Natural Products

Zhang et al. developed an extracellular inhibitor screening model and utilized this method to discover sodium tanshinone IIA sulfonate (STS), a sulfonate derived from tanshinone IIA (TSN), which has dual inhibitory effects on IDO1 and TDO2. Studies have demonstrated that STS can effectively reduce Kyn synthesis, inhibit tumor growth, and enhance the antitumor efficacy of PD1 antibodies. Furthermore, the combination therapy of STS and anti-PD1 has been shown to produce more effective outcomes in inhibiting tumor progression [30]. *Dactylicapnos scandens* (D. Don) Hutch. is a prominent species recognized for its ethnomedicinal applications. It is used for the treatment of inflammation and tumors [175,176]. The primary bioactive components of *D. scandens* are isoquinoline alkaloids (IQAs). Research has demonstrated that IQAs significantly inhibit the enzymatic activities of both IDO1 and TDO2. Bioassay-guided phytochemical investigation of Dactylicapnos scandens yielded a series of semi-synthetic tetrahydroisoquinoline alkaloids demonstrating dual inhibitory activity against IDO1 and TDO2, including dactycapnine A. The saturation transfer difference NMR spectroscopy (STD NMR) technique elucidated direct intermolecular interactions between dactycapnine A and both IDO1/TDO enzymatic targets. Furthermore, inhibitory kinetic analysis characterized dactycapnine A as a mixed-type inhibitor of both IDO1 and TDO2 [176]. Dactycapnine A is regarded as the most potent dual inhibitor of IDO1 and TDO2 within this series of tetrahydroisoquinoline alkaloids. However, this finding remains unsupported by corresponding cellular and animal experiments.

#### 5.2.3. Dual Inhibitors of IDO1/TDO2 Derived from Artificial Intelligence

Traditional new drug development encompasses the identification of lead compounds and subsequent optimization, a process that is characterized by its lengthiness, complexity, and high risk [177]. The rise of artificial intelligence has catalyzed a transformative shift in the field of new drug development. To date, artificial intelligence has developed numerous algorithmic models for drug discovery, including target protein structure identification [178], virtual screening [179], de novo drug design [180], and bioactivity and toxicity prediction [181]. Notably, virtual screening can be further categorized into ligand-based and structure-based approaches [177]. When the protein structure is available, structure-based virtual screening can be employed to identify hit compounds that serve as potential lead compounds and drug candidates [182,183,184]. Virtual hits must be evaluated through biological assays to confirm their binding affinity for the intended targets [185,186].

Suat et al. performed structure-based virtual screening of TDO2 using the ChEMBL collection and the IBS natural compound library. They conducted 3D shape similarity and pharmacophore modeling to screen approximately 67,000 natural products, ultimately identifying 30 virtual hits. They evaluated the function of these 30 virtual hits in inhibiting TDO2 by monitoring the changes in kynurenine in cancer cells in vitro. Finally, compound TD34 was selected. TD34 not only successfully inhibits the activity of TDO2 but also blocks the expression of IDO1, making it an effective dual inhibitor of IDO1/TDO2 [160]. Similarly, Naufa’s team identified five novel dual inhibitors of IDO1/TDO2 from the ZINC15 natural product library through consensus structure-based virtual screening, including ZINC00000079405/10, ZINC00004028612/11, ZINC00013380497/12, ZINC00014613023/13, and ZINC00103579819/14 [187]. Furthermore, a previous study reported virtual screening based on IDO1/TDO2 ligands. Based on the 12 hIDO1/hTDO2 dual inhibitor training set collected from previous studies, a ligand-based pharmacophore model was constructed. This model was applied to pharmacophore-based virtual screening. Based on the docking scores, compliance with the pharmacophore model, and binding affinity to the respective enzyme pockets, Pitavastatin, an anti-hypercholesterolemia drug, was ultimately selected as the most promising candidate [188]. Further studies have demonstrated that Pitavastatin can significantly inhibit the activities of IDO1 and TDO2 enzymes in vitro. In HepG2 cells, Pitavastatin can modulate cell cycle regulation, induce G1/S phase arrest, decrease caspase-3 activity, and promote apoptosis. Furthermore, it demonstrates significant cytotoxicity against BT-549, MCF-7, and HepG2 cell lines while maintaining a considerable safety margin in normal breast cells (MCF10-A) [188]. These findings underscore the substantial potential of Pitavastatin in cancer therapy. However, further studies, including both in vitro and in vivo experiments, are necessary to fully assess the efficacy and safety of these AI-designed drugs mentioned above.

**Table 3:** Application of targeting TDO2 in digestive system disease treatment.

Targeted Agents	Object	Molecules Targeted	Outcomes	Ref.
680C91	Primary normal human lung fibroblasts (NHLFs), CL1-5 human lung adenocarcinoma cell lines, and human lung cancer cells A549, mouse	TDO2	Improved T-cell response and DC function and decreased tumor metastasis	[162]
LM10	Mastocytoma P815, mouse	TDO2	Prevent the growth of P815 tumor cells, show obvious signs of toxicity to mice	[164]
Aminoisoxazoles	Rat, dog, and human whole blood, SW48 cells, A172 cells	TDO2	Inhibit the activity of TDO2	[165]
PF045102/ EOS200809	Colon carcinoma line CT26, MC38, mouse	TDO2	Improve the efficacy of checkpoint inhibitors	[166]
EPL-1410	Colon carcinoma line CT26, melanoma line B16F10, mouse	IDO1/TDO2	Reduce the levels of biomarkers (Kyn/Trp) in plasma, tumor-draining lymph nodes, and tumor tissue without eliciting any treatment-related adverse clinical symptoms or weight loss	[170]
AT-0174	Human non-small-cell lung cancer cells and mouse Lewis lung cells, mouse	IDO1/TDO2	Enhance antitumor immunity in platinum-resistant non-small-cell lung cancer	[29]
AT-0174	Glioma cell line GL261, mouse	IDO1/TDO2	Synergizes with temozolomide to improve survival in an orthotopic mouse model of glioblastoma	[171]
RY103	Glioma cell lines U87MG, U251, A172, and GL261, patient samples	IDO1/TDO2	Inhibit the migration, invasion, and growth of glioma cells	[172]
RY103	Pancreatic cancer cell lines KPIC, PANC1, and Pan02, mouse	IDO1/TDO2	Inhibit the migration and invasion of pancreatic cancer cells	[114]
RY103	Glioma cell line GL261, U87MG, U251, U251, mouse, patient samples	IDO1/TDO2	Suppress Trp-GCN2-mediated angiogenesis and counter immunosuppression in glioblastoma	[173]
M4112	Patient samples, mice with CT26-KSA tumors	IDO1/TDO2	Decrease the kynurenine: tryptophan ratio in the liver and tumor	[174]
Sodium tanshinone IIA sulfonate (STS)	Colon carcinoma line CT26, mouse	IDO1/TDO2	Inhibit tumor growth, combined therapy with STS plus anti-PD1 is more effective	[30]

## 6. Discussion

The incidence of digestive system diseases is high and increasing year by year, significantly impacting patients’ survival rates and diminishing their overall quality of life. The metabolism of tryptophan plays a crucial role in physiological processes. TDO2 serves as an indispensable rate-limiting enzyme in the initial stage of tryptophan metabolism and exerts a significant influence on the progression and prognosis of digestive system diseases. Aberrant expression of TDO2 is closely associated with the size of digestive system tumors, cancer staging, and disease prognosis. Consequently, a comprehensive investigation into the function and mechanism of TDO2 holds significant promise for the alleviation and treatment of digestive system diseases in the future. The development of effective drugs targeting TDO2 represents a crucial strategy for inhibiting the progression of digestive system tumors and inflammation, thereby enhancing patient prognosis. With advancements in research methodologies, the advent of computationally assisted drug design has substantially improved our capacity to discover novel therapeutics while simultaneously reducing the research and development timeline for targeted new drugs. Furthermore, by adopting a comprehensive treatment strategy that combines targeted inhibitors of TDO2 with immunotherapy, unexpected effects may be achieved in controlling the progression of digestive system diseases. However, the lack of a comprehensive understanding of the hTDO2 structure has posed significant challenges in identifying more potent TDO2 inhibitors. Presently, most research on TDO2 inhibitors remains at the laboratory investigation stage, and the precise mechanisms of action have yet to be fully elucidated. Substantial progress is still required before these inhibitors can be translated into clinical medication. Moreover, it is noteworthy that under normal physiological circumstances, TDO2 facilitates the catabolism of tryptophan, thereby playing a pivotal role in maintaining homeostasis. Current research suggests that the role of TDO2 varies across different diseases. For instance, TDO2 appears to exert a beneficial effect in conditions such as viral hepatitis and inflammatory bowel disease. Moreover, even within the same disease context, the function of TDO2 remains controversial. As mentioned earlier, the role of TDO2 in liver cancer is still subject to two diametrically opposed views. Furthermore, 3-hydroxykynurenine, the downstream product of TDO2, plays a role in safeguarding the eyes against ultraviolet damage, and Kyna exhibits neuroprotective properties. Thus, while inhibiting TDO2 could present an effective approach for addressing digestive system diseases as mentioned above, it may also produce unforeseen side effects. It is imperative to consider the potential safety concerns associated with TDO2 inhibition.

## 7. Conclusions

Aberrant expression of TDO2 has been implicated in various digestive system diseases, yet the precise mechanisms underlying its role remain to be fully elucidated. The inability of the IDO1-specific inhibitor to completely block the production of immunosuppressive tryptophan catabolites may contribute to the suboptimal clinical efficacy observed in its phase 3 trial. Therefore, exploring the use of combined inhibitors specific to IDO1 and TDO2, or administering dual inhibitors that target both IDO1 and TDO2, represents a promising strategy to more effectively block the immunosuppressive kynurenine pathway. Currently, the development of TDO2 inhibitors and dual IDO1/TDO2 inhibitors remains a formidable challenge. AI has shown enhanced outcomes throughout multiple stages of drug development. Nevertheless, substantial hurdles persist in this field.

## Data Availability

Not applicable.
